# Systematic review on molecular detection of congenital and neonatal infections caused by TORCH and SARS-CoV-2 in newborns’ cerebrospinal fluid

**DOI:** 10.1590/1984-0462/2025/43/2023191

**Published:** 2024-09-06

**Authors:** Suzana Ferreira Zimmerman, Sandra Helena Alves Bonon, Sergio Tadeu Martins Marba

**Affiliations:** a Universidade Estadual de Campinas, Campinas, SP, Brazil.

**Keywords:** Congenital toxoplasmosis, Congenital syphilis, Herpes simplex encephalitis virus, Congenital Zika syndrome, Polymerase chain reaction, Cerebrospinal fluid, Toxoplasmose congênita, Sífilis congênita, Vírus da encefalite herpes simples, Síndrome congênita de Zika, Reação em cadeia da polimerase, Líquido cefalorraquidiano

## Abstract

**Objective:**

To verify the use and identify advantages of molecular methods for congenital infections diagnosis in cerebrospinal fluid of neonates.

**Data source:**

The review was registered in the International Prospective Register of Systematic Reviews (PROSPERO), under CRD42021274210. The literature search was performed in databases: PubMed, Virtual Health Library/ Latin American and Caribbean Center on Health Sciences Information (VHL/BIREME), Scopus, Web of Science, Excerpta Medica database (EMBASE), Cochrane, ProQuest, and EBSCOhost. The search was carried out from August to October 2021 and updated in December 2022, respecting the Preferred Reporting Items for Systematic Reviews and Meta-Analyses (PRISMA) guidelines. The selection sequence was: 1) Duplicate title removal; 2) Examination of titles and abstracts; 3) Full-text retrieval of potentially relevant reports; and 4) Evaluation of the full text according to eligibility criteria by two independent authors. Inclusion criteria considered randomized and non-randomized control trials, longitudinal, cross-sectional, and peer-reviewed studies in humans, published in English, Spanish, Italian, and Portuguese, with newborns up to 28 days old who had congenital neuroinfections by toxoplasmosis, rubella, cytomegalovirus, herpes simplex (TORCH), and others such as *Treponema pallidum*, Zika, parvovirus B-19, varicella zoster, Epstein-Barr, and SARS-CoV2, diagnosed by polymerase chain reaction (PCR). Two evaluators extracted the following information: author, year of publication, nationality, subjects, study type, methods, results, and conclusion.

**Data synthesis:**

The most studied pathogen was herpes simplex. Several articles reported only nonspecific initial symptoms, motivating the collection of cerebrospinal fluid and performing PCR for etiological investigation.

**Conclusions:**

Molecular methods are effective to detect pathogen genomes in cerebrospinal fluid, which can impact clinical evolution and neurological prognosis.

## RESUMO

**Objetivo:**

Verificar a utilização e identificar as vantagens dos métodos moleculares para diagnóstico de infecções congênitas no líquido cefalorraquidiano de neonatos.

**Fontes de dados:**

A revisão foi registrada na base PROSPERO (*International Prospective Register of Systematic Reviews*) sob CRD42021274210. A busca bibliográfica foi realizada nas bases de dados PubMed, Biblioteca Virtual em Saúde/ Centro Latino-Americano e do Caribe de Informação em Ciências da Saúde (BVS/BIREME), Scopus, Web of Science, *Excerpta Medica database* (EMBASE), Cochrane, ProQuest, e EBSCOhost. A busca foi feita no período de agosto a outubro de 2021 e atualizada em dezembro de 2022, respeitando as orientações do *Preferred Reporting Items for Systematic Reviews e Meta-Analyises* (PRISMA). A sequência da seleção dos estudos foi: 1) Remoção de duplicatas; 2) Exame de títulos e resumos; 3) Recuperação dos textos completos potencialmente relevantes; e 4) Avaliação do texto completo conforme critérios de elegibilidade por dois autores independentes. O critério de inclusão considerou ensaios clínicos randomizados e não randomizados, estudos longitudinais, transversais, revisados por pares, estudos em humanos, publicados em inglês, espanhol, italiano e português, com recém-nascidos de até 28 dias que sofreram neuroinfecções congênitas pelos agentes toxoplasmose, rubéola, citomegalovírus, herpes simples (TORCH), e outros como *Treponema pallidum*, Zika, parvovírus B-19, varicela zoster, Epstein-Barr, e SARS-CoV-2, diagnosticadas por reação em cadeia de polimerase (PCR). Dois avaliadores extraíram as seguintes informações: autor, ano de publicação, nacionalidade, sujeitos, tipo de estudo, métodos, resultados e conclusão.

**Síntese dos dados:**

O patógeno mais estudado foi Herpes Simples. Muitos artigos relataram somente sintomas iniciais inespecíficos, motivando a coleta de líquido cefalorraquidiano e realização da PCR para investigação etiológica.

**Conclusões:**

Os métodos moleculares são eficazes para detectar o genoma do patógeno no líquido cefalorraquidiano, o que pode impactar na evolução clínica e no prognóstico neurológico.

## INTRODUCTION

The risk of transmission of congenital infections from mother to fetus depends on the type of infection and the time of gestation. Generally, the most severe conditions are acquired in the first trimester of pregnancy.^1-3^ Some of these infections are caused by TORCH agents: toxoplasmosis, rubella, cytomegalovirus, herpes simplex types 1 and 2 and others, such as syphilis, varicella zoster, parvovirus B-19, SARS-CoV-2, and Zika virus.^4-9^ The consequences of this can be stillbirths, prematurity, uterine growth retardation, and congenital malformations.^10-16^

These consequences are known; therefore, there must be fast and sensitive diagnostic methods in order to change the prognosis of these newborns. Detection of deoxyribonucleic and ribonucleic acids (DNA/RNA) in cerebrospinal fluid (CSF) by molecular methods, such as polymerase chain reaction (PCR), is a marker of the congenital infection involvement in the nervous system.^17-23^

This review aimed to verify the use and advantages of molecular methodologies (PCR, nested-PCR, real-time PCR) to detect TORCH and coronavirus disease 2019 (COVID-19) pathogens in CSF of newborns aged from one to 28 days old, with suspected congenital infection.

## METHOD

This is a systematic review about congenital infections diagnosed in the nervous system by molecular biology using the PCR technique registered in the International Prospective Register of Systematic Reviews (PROSPERO) database, number CRD42021274210.

The literature search was performed in the following main databases: PubMed, Virtual Health Library of the Latin American and Caribbean Center on Health Sciences Information (VHL/BIREME), Scopus, Web of Science, Excerpta Medica database (EMBASE), Cochrane, ProQuest, and EBSCOhost. There was no restriction on publication dates. We included published articles in English, Spanish, Italian, and Portuguese. The search was carried out from August to October 2021 and updated in December 2022.

The detection of TORCH and COVID-19 congenital infections in CSF by molecular methods was reviewed, using specific descriptors and their synonyms according to the Medical Subject Headings (MeSH) and Health Sciences Descriptors (*Descritores em Ciências da Saúde,* DeCS). Search terms were “newborns” or “neonates” and TORCH agents: “congenital syphilis” or “congenital toxoplasmosis” or “varicella-zoster virus encephalitis” or “parvovirus B-19” or “congenital rubella syndrome” or “congenital cytomegalovirus” or “herpes simplex virus encephalitis” or “Epstein Barr virus infection” or “congenital Zika” or “SARS-Cov-2” or “COVID-19” and “PCR” or “polymerase chain reaction” and “cerebrospinal fluid”. In this research, Boolean operators and symbols such as OR and AND were used to compose a search string.

The review followed the checklist criteria described by the Preferred Reporting Items for Systematic Reviews and Meta-Analyses (PRISMA).

In the papers’ selection process, the following sequence was respected:

1. Duplicate title removal;
2. Examination of titles and abstracts;
3. Full-text retrieval of potentially relevant reports; and
4. Evaluation of the full text potentially qualified study and assessment of the eligibility criteria.

Two authors independently screened the studies by title and abstract for potential inclusion in this review. Full texts of all selected articles were subsequently reviewed and assessed for eligibility. Disagreements or conflicts between reviewers were resolved by consensus.

**Figure 1:**
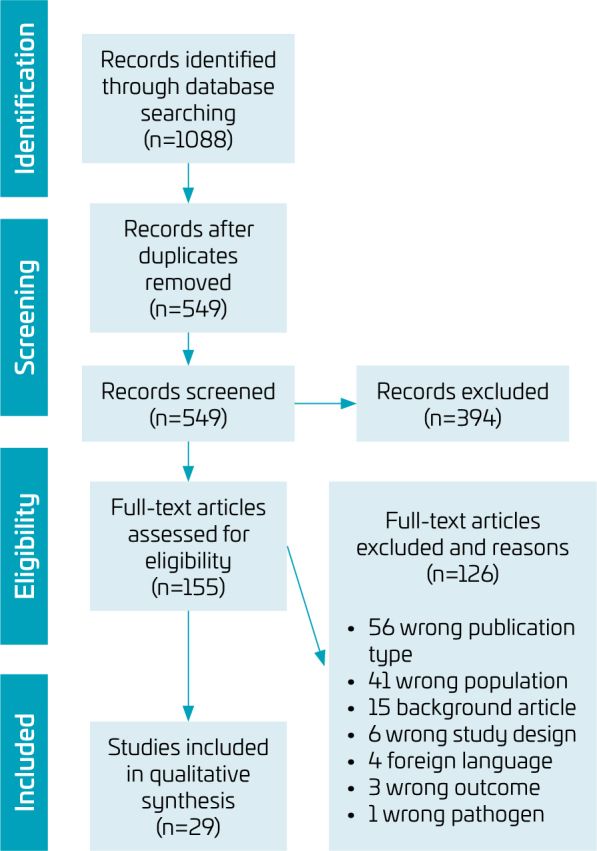
Flowchart for studies’ selection.

Manual searches were performed on the references of the studies included in the final review process. Information on the different phases of the selection process was carried out based on the PRISMA flow diagram (Figure 1).

Randomized controlled trials (RCTs), longitudinal studies (retrospective, prospective, case-control), cross-sectional studies, and non-RCTs with newborns up to 28 days old who had congenital neuroinfections (by TORCH and SARS-CoV-2 agents) were included once the diagnosis of congenital infections had been investigated by PCR-based techniques applied to neonatal CSF analysis. Exclusion criteria were: papers with only abstracts (due to limited information); those on therapeutics; editorials; commentaries; unpublished manuscripts; guidelines (usually based on non-research evidence or expert opinion); reviews and systematic reviews; meta-analyses of experimental and controlled observational studies; electronic websites; conference proceedings, dissertations and PhD thesis, research reports; animal studies; case series (because of the small number of patients included in these articles). Studies with newborns up to 28 days old who underwent other diagnostic methodologies of congenital infections by TORCH agents or enrolled infants aged 29 days and older were also excluded. The final decision was made after a consensus among all the authors.

Two evaluators performed data extraction and analysis of the selected articles. They extracted the necessary information such as authors, year of publication, nationality, study design and type, subjects, and more detailed information about molecular methodology for deoxyribonucleic and ribonucleic acids (DNA/RNA) detection of TORCH and SARS-CoV-2 pathogens causing congenital infections in CSF, and results and conclusions of the selected studies. This data was organized in the Microsoft Office Excel 365 software.

## RESULTS

Data extracted from the included articles are summarized according to etiological agents of congenital neuroinfections in Table 1, *Herpesviridae* family agents (herpes simplex virus [HSV-1 and HSV-2], varicella zoster [VZV], Epstein-Barr virus [EBV], cytomegalovirus [CMV]) and parvovirus B-19. In Table 2, Toxoplasmosis; in Table 3, Zika virus (ZIKV); and in Table 4, *Treponema pallidum* (syphilis pathogen).

**Table 1:** Data synthesis from the articles about congenital Herpesviridae infections in the review.

Study	Country, Year	Subjects	Study type	Methods	Results and Conclusion
Anderson et al.^1^	New Zealand, 1993	Patients with clinical HSV encephalitis	prospective	detection of HSV DNA with PCR in CSF	PCR is a highly specific test for HSV-1 and HSV-2 encephalitis in newborns
Czech-Kowalska et al.^5^	Poland, 2021	168 infants with cCMV	retrospective and prospective	retrospective (2012–2015) and prospective (2016–2019)	PCR in CSF: 23 positive for CMV, all symptomatic
Davis et al.^6^	USA, 2008	infants 0–60 days; 88 cases and 83 controls	case-control study	PCR in CSF; 1999–2004	HSV: 3,4% by PCR; seizure associated with decision to CSF HSV PCR
Jeyanthi et al.^13^	Singapura, 2015	neonates with HSV in CNS	retrospective	PCR of HSV in CSF; 1998–2011	33% seizures, 44% vesicles
Kawada et al.^14^	Japan, 2004	Neonates with HSV encephalitis	prospective	real-time PCR HSV compared with nested PCR in 109 CSF	Real-time PCR sensitivity: 100%; specificity: 99%
Kleines et al.^15^	German, 2014	100 newborns, 2001–2012	retrospective	CSF PCR of HSV, VZV, EBV, CMV	Neonates: only CMV detected (4%)
Kurtz et al.^16^	United Kingdon, 2003	infants with HSV encephalitis	retrospective	CSF: HSV PCR were reviewed, follow-up 1996–1999	HSV PCR should be done in all neonates with encephalopathy
Malm and Forsgren^17^	Sweden, 1999	36 neonatal HSV infections	retrospective	PCR in CSF; 1973–1996	in CNS: 29; seven had HSV-1 and 29 HSV-2
Melvin et al.^20^	USA, 2015	neonates with HSV in CNS	retrospective	medical record review 1993–2012	HSV PCR in CSF: 70% of neonates with encephalitis
Mustonen et al.^23^	Finland/ UK, 2003	Neonates with seizures	prospective	PCR for: HSV-1 and 2, VZV 1995–1997	Only HSV-2 found in CSF samples of these neonates
Norero^24^	Panama, 2015	febrile (60 neonates)	prospective descriptive	PCR of CSF; 2013 – 2014	2% CMV infection; none HSV in suspicion of sepsis
Parisi et al.^26^	Italy, 2016	68 newborns, 2012 - 2015	retrospective	PCR for HSV/EBV/VZV/CMV/PvB-19	None of these pathogens detected in neonates’ CSF
Petel et al.^27^	Canada, 2020	younger than 90 days of age	cross-sectional retrospective	PCR in CSF, 2013–2014	Viral CNS infections more than bacterial in infants
Schleede et al.^29^	German, 2013	Neonates with encephalitis	retrospective and prospective	HSV encephalitis diagnosed by PCR	difficulty to diagnose because of nonspecific early symptoms

HSV: herpes simplex virus; DNA: deoxyribonucleic acid; PCR: polymerase chain reaction; CSF: cerebrospinal fluid; CMV: cytomegalovirus; cCMV: congenital cytomegalovirus; USA: United States of America; CNS: central nervous system; VZV: varicella zoster virus; EBV: Epstein-Barr virus; UK: United Kingdom.

**Table 2:** Data synthesis from articles about congenital toxoplasmosis included in the review.

Study	Country, Year	Subjects	Study type	Methods	Results and Conclusion
Belaz et al.^2^	France, 2015	41 suspicious congenital toxoplasmosis	Observational, longitudinal retrospective	PCR diagnosis from 2003 to 2013. All positive samples were reanalyzed with both gene targets in the same PCR. All samples from proven congenitally infected infants.	PCR positive for congenital toxoplasmosis in CSF and suggest that region should be adopted as part of the standardization
Cassaing et al.^4^	France, 2006	prenatal and neonatal diagnosis of toxoplasmosis	prospective study	Between June and October 2003, all samples for suspicion were tested	PCR method: sensitivity was confirmed in positive clinical samples including CSF (50%) PCR improved the performance of the diagnosis of toxoplasmosis
Olariu et al.^25^	USA, 2014	infants ranged in age from 0–180 days, born from untreated mothers	retrospective	PCR in CSF for diagnosis of congenital toxoplasmosis. Studied infants, diagnosed clinically and serologically, noninfected, between 1993–2005	PCR in CSF: sensitivity varied by age group: 0–30 days = 60% VPP and specificity: 100% CSF PCR can contribute to the early confirmation of the diagnosis of congenital toxoplasmosis

PCR: polymerase chain reaction; CSF: cerebrospinal fluid; USA: United States of America; VPP: positive predictive value.

**Table 3:** Data synthesis from the articles about congenital Zika infection included in the review.

Study	Country, Year	Subjects	Study type	Methods	Results and Conclusion
Araújo et al.^7^	Brazil, 2016	32 case patients (neonates with microcephaly) and 62 controls (neonates without microcephaly)	case-control study	January to May 2016 CSF samples of cases were tested for Zika virus by RT-PCR. Laboratory-confirmed Zika virus infection a positive RT-PCR result in neonates	80% mothers of cases had Zika virus infection while 64% mothers of controls 41% cases and none of controls had laboratory-confirmed Zika virus infection. Microcephaly epidemic is a result of congenital Zika virus infection
Araújo et al.^8^	Brazil, 2018	173 controls, 91 cases of neonates with microcephaly	case-control study	January to November 2016 CSF samples of cases tested for Zika virus, specific IgM and by quantitative RT-PCR	Cases: 43% positive for Zika virus and had cerebral abnormalities Association between microcephaly and congenital Zika virus infection was confirmed
Pomar et al.^28^	French Guiana, 2018	305 newborns/fetuses from pregnant women with a laboratory-confirmed Zika virus infection during the epidemic period in French Guiana.	prospective	January to July 2016 Rate of maternal-fetal transmission of Zika virus CSF, signs of congenital Zika syndrome, severe complications or fetal loss. Associations between laboratory-confirmed infection and adverse fetal/neonatal outcomes	Positive Zika virus results were obtained from (57%) CSF. Maternal-fetal transmission seems to occur in approximately 1/4 of exposed fetuses and is associated with early adverse fetal/neonatal outcomes in 1/3 of infected fetuses

CSF: cerebrospinal fluid; RT-PCR: real-time PCR; PCR: polymerase chain reaction.

**Table 4:** Data synthesis from the articles about congenital syphilis included in the review.

Study	Country, Year	Subjects	Study type	Methods	Results and Conclusion
Marangoni et al.^18^	Italy, 2011	165 newborn to 151 syphilis seropositive mothers with + RPR at birth	prospective surveillance	CSF analysis PCR, 2000 through 2010 prospective follow up	careful CSF examination allowed the identification and treatment of high-risk newborns
Marangoni et al.^19^	Italy, 2008	133 infants Born to 119 syphilis seropositive mothers	prospective	six presumptive cases of congenital syphilis with IgM western blot positive results were analysed CSF, from 2000 through 2007	careful CSF examination allowed the identification and treatment of high-risk newborns
Michelow et al.^21^	USA, 2002	148 infants were included whose mothers had syphilis during pregnancy	prospective	prospectively enrolled if born between 1989 and 1999	*Treponema pallidum* detected in CSF in 22% who had no prior antibiotic exposure. CNS infection was best predicted by IgM of serum or PCR of serum or blood. Only 35% of these infants had positive CSF PCR.

RPR: rapid plasma regain; CSF: cerebrospinal fluid; PCR: polymerase chain reaction.

## DISCUSSION

Infections in the central nervous system (CNS) of neonates may be caused by several etiological agents. In the literature, we found that 66% of encephalitis is caused by viruses^24-27^ and 26% of newborns who present with seizures have some neuroinfection of viral etiology.^23^

The differential diagnosis of congenital neuroinfections only by clinical signs is quite difficult, due to the presentation of nonspecific signs in this age group, that are not pathognomonic of specific pathogens.^26-28^ It is, therefore, important to identify the pathogen in the CSF, which can impact the evolution and the neurological prognosis of these newborns, since molecular methods are fast and effective for detecting these causal agents, allowing the early institution of the recommended therapy.^29^

The discussion will focus on each pathogen separately and the studies that investigated it.

### Herpes simplex

The most frequent among the congenital infections studied in the CSF found in this review was the neuroinfection caused by HSV (16/29, 55%). HSV-1 and 2 were the most common pathogens isolated by PCR performed in neonatal CSF. Acute sporadic encephalitis is most frequently caused by HSV-1 and 2^1,3,6,9^ in patients aged 0–19 years, accounting for about 31–50%^15,16^ of all herpetic encephalitis. While HSV type 1 is normally isolated from adults and children, HSV type 2 is detected mainly in newborns.^17,20^

Vertical transmission of HSV usually occurs during childbirth as a result of contact with the maternal infectious secretion during passage through the vaginal canal; however it can also occur through the transplacental and postnatal route, but does not contraindicate breastfeeding.^23,26^ This predominant mode of transmission in the birth canal should explain why HSV-2 predominated over HSV-1 in newborn CSF.^1,12,13,16,17^ HSV-1 and 2 can cause different morbidities and establish latent infections that can be further reactivated, causing lesions located at the primary site of infection or close to it.

About 30% of infected neonates have HSV in the CNS and the symptoms are usually nonspecific, similar to a severe bacterial infection. Skin lesions are an indication of HSV infection; however, 35% of newborns do not manifest this symptom.^27^ Herpetic encephalitis can have a devastating course, with a poor prognosis. Approximately 70% of untreated patients die. Meningitis occurs in 10% of cases of primary HSV-2 infection.

In several studies included in this review,^6,14,16,26,29^ there are reports of only nonspecific initial signs, while Mustonen et al.^23^ showed that the incidence of CNS viral infections with neonatal seizures must be much higher than previously reported. Another article mentioned seizures in more than 50% of newborns, motivating the collection of CSFs and the performance of PCR to investigate the causal agent. Petel et al.,^27^ in 2020, studied infants younger than 90 days of age, hospitalized with HSV CNS infection at seven pediatric academic centers (a Paediatric Investigators’ Collaborative Network on Infections in Canada - PICNIC) and found that ages younger than 21 days and the presence of seizures or extra-CNS involvement are clues to HSV infection.

The sensitivity of HSV-1 and 2 in the CSF of neonates with disease in the CNS detected by PCR, according to some authors, is around 70%,^23,24,26^ and it is consistent with the pathogenetic model that indicates that encephalitis occurs before meningitis^20,27,29^. According to Jeyanthi et al.,^13^ the initial PCR for CSF HSV was positive in 78% (HSV-2 is the most common). A repeat CSF study showed HSV positivity of 100%. Diagnosis by PCR is of great value in identifying the type of herpes virus in cases of herpetic encephalitis.^27^

Norero,^24^ in a study performed in Panama with febrile neonates suspected of sepsis, concluded that PCR in CSF can lead to less hospital expenses (fewer days staying hospitalized) and rational use of antibiotics.

### Varicella zoster

Primary infection with VZV or human herpesvirus 3 (HHV-3) during pregnancy brings significant complications for maternal and fetal health. If the mother acquires varicella infection during the early gestational period (weeks 8 to 20), the fetus is at risk of developing congenital varicella syndrome.^3,15^

Congenital varicella syndrome is characterized by an embryofetopathy, which includes cicatricial skin lesions, limb hypoplasia, muscle atrophy, clubfoot, intrauterine growth restriction, microcephaly, cerebellar and cortical atrophy, hydrocephalus, seizures, and intracranial and extracranial calcifications.^26^ With neurological involvement, the following may occur: cerebellar ataxia (usually the first sign of encephalitic involvement); acute myelitis and optic neuritis; polyradiculoneuritis; CSF with an eventual increase in lymphocytic cellularity; meningoencephalitis; and seizures.

Among the studies included in the review with molecular analysis of the CSF by PCR for pathogens that cause congenital infections, there were four that investigated, beyond the presence of HSV-1 and 2, the VZV,^3,15,23,26^ and other agents of the *Herpesviridae* family, such as CMV and the EBV. There were no positive PCR for VZV in CSF samples of neonates in these studies.

### Epstein-Barr Virus

EBV is also called human herpesvirus 4 (HHV-4). The transmission can occur through saliva, but also through the transplacental route (vertical transmission). During the first trimester of pregnancy, it can infect the fetus, causing a syndrome with several congenital anomalies (micrognathia, cryptorchidism, and cataracts), hypotonia, thrombocytopenia, persistent monocytosis, proteinuria, and metaphysitis at birth. The presence of this agent was investigated in studies that looked for several pathogens of the *Herpesviridae* family by Molecular Biology in the CSF of neonates.^3,15,26^ None of them found EBV by PCR in the CSF of the studied neonates.

### Cytomegalovirus

CMV is the human herpesvirus 5 (HHV-5) that is usually found in the urine and saliva of infected people, intermittently, but can also be found in the CSF, as well as in the mucus of the uterine cervix, semen, feces, and breast milk. Therefore, it can be transmitted sexually, non-sexually, and vertically (from mother to child). It is known as the most frequent cause of congenital infections in the world.^3,5,10,11,15,24,26^

Perinatal infection is asymptomatic in most full-term newborns.^10^ However, it may be associated with clinical conditions of varying severity, such as the “sepsis-like” syndrome, cholestasis, thrombocytopenia, neutropenia, and pneumonitis, when it affects preterm newborns weighing less than 1.500g and/or gestational age of less than 32 weeks.^11,15^ The involvement of the CNS must be evaluated with special attention in the presence of sensorineural deafness and, neuropsychomotor development delay. In this review, 7/29 (24%) studies identified this pathogen in CSF by PCR.^3,5,10,11,15,24,26^

Czech-Kowalska et al.^5^ performed PCR in CSF and found 145 negative (6 asymptomatic and 139 symptomatic) results and 23 positive for CMV (all symptomatic), concluding that there was a higher rate of CNS damage associated with positive PCR.

### Syphilis

Syphilis is a systemic infection caused by the spirochete *Treponema pallidum*, which is of particular concern during pregnancy due to the risk of transplacental infection. Congenital infection is associated with several adverse outcomes. When *Treponema pallidum* invades the CNS, it can cause varied clinical conditions, from alterations in CSF in asymptomatic patients, to more serious conditions such as progressive general paralysis.^18,19^

Molecular tests show better results than serological tests, with a sensitivity of 91% for diagnosing syphilis. However, due to their high cost and complexity of performing, they are not widely distributed to routine laboratories. In contrast, serological, treponemal, and non-treponemal tests are used in clinical practice for the increasing prevalence of this pathology in pregnant women, with PCR being limited to research centers.^21^ This review included three articles with CSF analysis identifying *Treponema* by molecular tests.^18,19,21^ In two of these studies,^18,19^ presumptive cases of congenital syphilis with positive Western Blot IgM results were found. Of these, only some had CSF with positive PCR for *Treponema*. These observations confirmed that prenatal syphilis screening facilitates treatment during pregnancy and reduces the risk of mother-to-child transmission. In addition, the use of IgM Western Blot and careful examination of CSF allowed the identification and treatment of high-risk newborns. In these studies, PCR was less sensitive because it detected fewer cases than serologic tests.^18,19,21^ In another study, only 35% of these children had positive CSF syphilis identified by PCR.^21^ On the other hand, 13 of 14 children with positive PCR for syphilis in CSF (93%) had positive serum IgM immunoblotting results.

*Treponema pallidum* infection of the CNS can be identified in most children by physical examination, standard laboratory tests, and radiographic studies.

### Toxoplasmosis

Toxoplasmosis is a disease caused by the protozoan *Toxoplasma gondii* that has a wide geographic distribution and high serological prevalence, up to 60% of the population in certain countries.^2^ Congenital toxoplasmosis may occur when the mother acquires an acute infection during pregnancy and consequences for the fetus will depend on factors such as degree of exposure of the fetus and period of gestation.^4^ In this review, three studies analyzing this agent were identified.^2,4,25^

In one study, the PCR performed was positive in CSF, with sensitivity in the neonates’ group of 60%. In newborns of mothers who were not treated for toxoplasmosis during pregnancy, CSF PCR can contribute to the early confirmation of the diagnosis of congenital toxoplasmosis, especially in those with clinical signs.^25^

### Parvovirus B-19

Transmission of parvovirus B-19 occurs through secretions from the respiratory system or vertical transmission in 30% of cases of maternal infection. The risk of transplacental transmission is highest in the first and second trimesters of pregnancy. Laboratory tests, such as immunoglobulins IgG, are necessary, especially when the mother presents with skin rash. However, there are cases of asymptomatic infections that do not exclude transmission to the fetus. In this review, an article was found analyzing the CSF of 29 neonates by PCR for this pathogen but there were no positive samples.^26^ Detection rate was low, probably because it was an unselected cohort.

### Zika virus

ZIKV is an arbovirus that belongs to the *Flaviviridae* family. In 2015, the circulation of the ZIKV was confirmed in Brazil, as well as the acute exanthematous disease caused by this etiological agent, and microcephaly in newborns who acquired this infection through vertical route.^7,8^ Other signs of this congenital infection (calcifications, ventriculomegaly, and cortical development disorder) made up the congenital Zika syndrome,^28^ whose epidemic was established in 2015, and 2,205 cases were confirmed by the end of 2016.^7,8^

In this review, three articles^7,8,28^ were included with the identification of the ZIKV as a pathogenic agent of the CNS in CSF identified by PCR.

Two of these studies were case-controls. The cases included newborns with microcephaly and controls were neonates without microcephaly. Mothers of newborns with microcephaly (cases) had more serological markers of previous ZIKV infection compared to mothers of controls (without microcephaly), although mothers in both groups were PCR negative.^8^ Among newborns, 33 of 91 cases (35%) and 173 control infants had no laboratory-confirmed ZIKV infection. Among the 23 neonates that tested positive for ZIKV, there were 10 that had brain abnormalities (43%) and 13 that had no brain abnormalities, while 11 of 56 (20%), that were laboratory-confirmed negative for ZIKV, had brain abnormalities. The association between microcephaly and congenital ZIKV infection has been confirmed.^8^

In another study, maternal-fetal transmission appears to occur in approximately 1/4 of exposed fetuses and is associated with adverse fetal and early neonatal outcomes in 1/3 of infected fetuses: fetal loss or severe signs of congenital Zika syndrome.^28^

### SARS-CoV-2

In this review, one study presented patients who met the criteria of the Centers for Disease Control and Prevention (CDC) for confirmed COVID-19 infection and underwent lumbar puncture in the same admission.^22^ Among patients diagnosed with SARS-CoV-2, there were newborns with CSF results that changed treatment decisions, either by adjusting antibiotics, influencing therapeutic decision, or providing an alternative diagnosis. The real-time PCR (RT-PCR) in the CSF for SARS-CoV-2 was performed in 30% of patients, with negative results in all samples. More studies are needed to define whether CSF SARS-CoV-2 PCR is recommended in certain clinical settings.^22^

In the general context of congenital infections, there is a lack of information regarding PCR in CSF samples in the literature. This finding can be explained by one of two reasons: either it is a neglected subject that deserves more investigation, or it reflects that, in general, neonatologists do not suspect of CNS involvement in neonates presenting with congenital infections.

However, it is well known that the detection of pathogens in CFS is limited in daily care. The detection rate of pathogens in the CSF is low, even using molecular methods in infected infants with confirmed involvement of the CNS by neuroimaging. In consequence, a negative result in CFS by PCR does not rule out CNS damage in congenital infections. Despite this fact, PCR is relevant to identifying the pathogens in congenital neuroinfections, being herpes simplex the most studied agent.
